# Preoperative imaging biomarkers combined with tap test for predicting shunt surgery outcome in idiopathic normal pressure hydrocephalus: a multicenter retrospective study

**DOI:** 10.3389/fnagi.2025.1509493

**Published:** 2025-02-27

**Authors:** Wei Gao, Wei Liu, Yuqi Ying, Qingze Zeng, Jiadong Wang, Jingquan Lin, Xinxia Guo, Hongjie Jiang, Zhe Zheng, Zhoule Zhu, Junming Zhu

**Affiliations:** ^1^Department of Neurosurgery, The Second Affiliated Hospital, Zhejiang University School of Medicine, Hangzhou, Zhejiang, China; ^2^Clinical Research Center for Neurological Diseases of Zhejiang, Hangzhou, China; ^3^Department of Neurosurgery, Changxing people’s Hospital, Changxing, Zhejiang, China; ^4^Department of Neurosurgery, The Fourth Affiliated Hospital, Zhejiang University School of Medicine, Yiwu, Zhejiang, China; ^5^Department of Radiology, The Second Affiliated Hospital, Zhejiang University School of Medicine, Hangzhou, Zhejiang, China; ^6^Department of Neurosurgery, The Second Affiliated Hospital of Jiaxing University, Jiaxing, Zhejiang, China

**Keywords:** idiopathic normal pressure hydrocephalus, neuroimaging, shunt surgery, imaging biomarkers, tap test

## Abstract

**Objectives:**

The study aims to investigate the predictive performance of preoperative imaging features combined with tap test for the outcomes of ventriculoperitoneal (VP) shunt in idiopathic normal pressure hydrocephalus (iNPH).

**Methods:**

In this multicenter retrospective study, 166 iNPH patients who underwent VP shunt surgery between August 2019 and November 2023 were included. Preoperative clinical characteristics and imaging features were collected. Preoperative clinical assessment and at least 3 months of postoperative follow-up were performed. Multivariable logistic regression, sensitivity, specificity, and the area under the receiver operating characteristic curve (AUC) were used to evaluate predictive performance.

**Results:**

Out of 166 total patients, 96 were responders and 70 non-responders. The tap test showed significant difference between two group (*p* < 0.01). Multivariable logistic regression identified that a positive disproportionately enlarged subarachnoid space (DESH) sign (OR = 0.09, 95% CI: 0.04–0.22, *p* < 0.001) and a sharper callosal angle (CA) (OR = 0.97, 95% CI: 0.95–1.00, *p* = 0.02) were associated with symptom improvement after shunt. The sensitivity, specificity, and AUC of tap test were 0.64, 0.60, and 0.62, respectively. Combining CA and the tap test increased sensitivity to 0.85, while combining DESH, CA, and the tap test improved specificity and AUC to 0.67 and 0.72, respectively.

**Conclusion:**

The findings suggest that the imaging features DESH and CA, when combined with the tap test, enhance the prediction of VP shunt outcomes in iNPH patients. Despite the improved predictive capability, further research focusing on innovative biomarkers for VP shunt is warranted.

## Introduction

Idiopathic normal pressure hydrocephalus (iNPH) is a treatable neurological disorder characterized by enlarged ventricles, normal lumber cerebrospinal fluid (CSF) pressure, and a clinical triad including gait disturbance, cognitive impairment, and urinary dysfunction (Wikkelsø et al., 2013). iNPH is widespread in the elderly population, particularly prevalent among individuals aged ≥ 60, with an estimated occurrence of 1.3% (Martín-Láez et al., 2015). The symptoms deteriorate without timely and suitable treatment (Andrén et al., 2014). CSF shunting surgery stands as the sole clinical treatment option, with ventriculoperitoneal (VP) shunt being the most commonly used approach. A comprehensive analysis of multiple research studies reveals a significant variability in outcome of shunting surgery for iNPH, with the improvement rates ranging from 26% to 90% over a follow-up of 1–10 years (Miyajima et al., 2016; Wu et al., 2020; Junkkari et al., 2021). Identifying responders in iNPH patients for shunting surgery still remains a significant clinical challenge.

The tap test, which involves a lumbar puncture with removal of 30–50 ml CSF and clinical evaluation before and after the lumbar puncture, is primary used for screening responder candidates (Rydja et al., 2021). Mihalj et al. (2016) conducted a systematic review highlighting that the tap test demonstrates a very high positive predictive value of 92% (range: 73%–100%) but a notably low negative predictive value of 37% (range: 18%–50%). Also, it exhibits relatively high specificity (75%, range: 33%–100%) alongside comparatively low sensitivity (58%, range: 26%–83%). Shunt responders would be missed if the criteria is only dependent on tap test (Wikkelsø et al., 2013; Walchenbach and Geiger, n.d.).

Preoperative imaging features are commonly utilized by physicians to diagnose possible and probable iNPH patients, encompassing metrics such as Evans Index (EI), the Disproportionately Enlarged Subarachnoid Space Hydrocephalus (DESH), and Callosal Angle (CA) (Park et al., 2021; Subramanian et al., 2021; Chen et al., 2022; Pyrgelis et al., 2022). It remains uncertain whether these imaging features can predict the outcome of VP shunt surgery. The DESH, in particular, is implemented to improve the prediction of a positive shunt response as reported (Mori et al., 2012; Shinoda et al., 2017). Craven et al. (2016) demonstrated that a positive DESH outcome effectively predicted shunt responsiveness, with a sensitivity of 30.77% and a specificity of 72%. Additionally, the positive predictive value and negative predictive value were 77.42% and 25%, respectively. Several other imaging features have also been independently or jointly demonstrated as potential predictors of shunt surgery outcomes, although the conclusions remain uncertain (Carlsen et al., 2022).

Neither standalone imaging features nor the tap test showed satisfactory predictive accuracy for VP shunt outcomes. Combining the two approaches may improve both sensitivity and specificity. There is limited study researching on how to combine the two and integrate them as a novel indicator to predict the outcomes of VP shunt, which potentially optimize the diagnosis of iNPH and guide surgical decision-making. The objective of this multicenter retrospective study is to explore the predictive performance of combining imaging features and tap test for predicting the outcome of VP shunt surgery, and improve the senstivity solely based on tap test results.

## Materials and methods

### Patients and diagnosis

**FIGURE 1:**
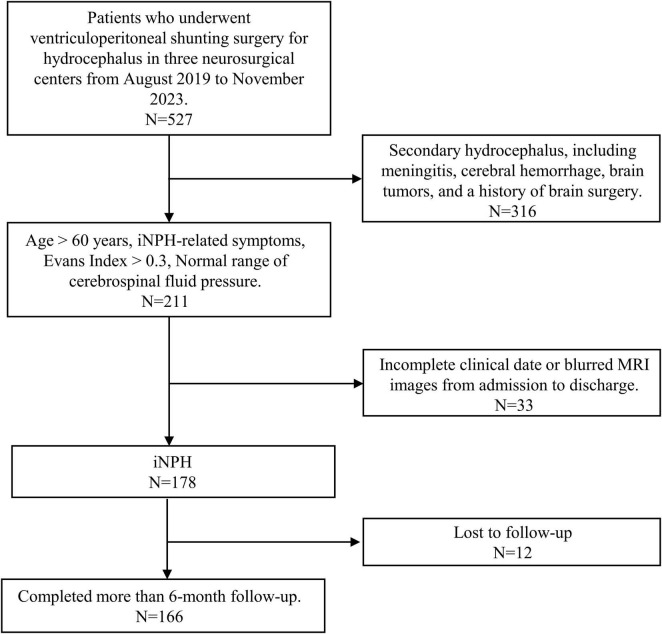
Flow chart depicting patients screening process for the = idiopathic normal pressure hydrocephalus (iNPH).

We conducted a multicenter retrospective analysis involving 166 iNPH patients who underwent ventriculoperitoneal shunting surgery in three neurosurgical centers, China, from August, 2019, to November, 2023. The screening process of the participants was illustrated in Figure 1. Clinical characteristics, including age, gender, tap test result, symptoms, duration of symptoms, lumbar puncture pressure, and comorbidities were collected. The diagnostic criteria were based on the second edition of the Japanese iNPH Treatment Guidelines, encompassing the following criteria: (1) Age > 60 years; (2) Presence of any one of the following symptoms: gait disturbance (mainly characterized by a short stride, shuffling gait, unsteady walking, and difficulty in turning), cognitive impairment, or urinary dysfunction; (3) Radiological evidence of ventricular enlargement, with an Evans Index > 0.3; (4) Normal range of cerebrospinal fluid pressure (80–200 mmH_2_O); (5) The above symptoms cannot be fully explained by other diseases (such as Alzheimer’s disease, Parkinson’s disease, cerebral small vessel disease, frontotemporal dementia, neurosyphilis infection, etc.); (6) The absence of other secondary factors causing hydrocephalus, such as obstructive hydrocephalus, cerebral hemorrhage, brain tumors, or a history of cranial surgery. Exclusion criteria were as follows: (1). All possible causes of secondary hydrocephalus, including meningitis, cerebral hemorrhage, brain tumors, and a history of brain surgery; (2). Blurred magnetic resonance imaging (MRI) images that could influence the calculation of the imaging index. The study was approved by three Hospitals of the ethics committee.

### Clinical assessment

Before the diagnostic tap test, gait was assessed using the 10 m walk test; Cognition was assessed with the mini-mental state examination; Urinary incontinence was subjectively assessed by patients on a scale from 0 to 10, indicating the severity from normal to severe. An identical assessment was performed 24–72 h later following tap test. Tap test positivity was defined based on the Japanese iNPH guidelines and other literatures (Nakajima et al., 2021; Pyrgelis et al., 2022), using the following criteria: (a) ≥ 20% improvement in time or steps of the 10 m timed walk test and/or (b) ≥ 10% improvement in MMSE score and/or (c) an improvement of ≥ 1 point in Urinary Incontinence score. Patients were classified as tap test positive if they met at least one of these criteria; otherwise, they were categorized as tap test negative.

According to preoperative evaluations, patients meeting the criteria for “probable iNPH” as defined by the Japanese guidelines are eligible for VP shunt surgery. For patients diagnosed with “possible iNPH,” surgical intervention may also be considered in cases of progressive symptom exacerbation, strong family preference, and a positive Continuous CSF Drainage Test.

The outcomes of the VP shunt were evaluated using the Krauss index at a follow-up of 6–12 months, where responders were defined as those with a Krauss index ≥ 0.5.

### Imaging features

**FIGURE 2:**
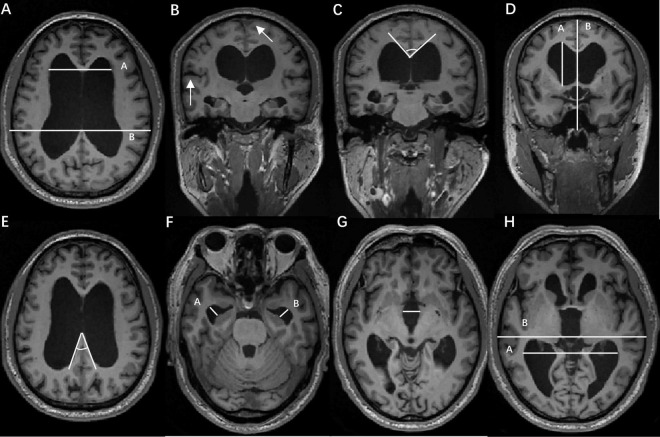
Radiological characteristics. **(A)**. Evans Index (EI), the maximum width of the frontal horns of the lateral ventricles to the maximal internal diameter of the skull at the same level, as measured in axial magnetic resonance imaging (MRI) images; **(B)**. Disproportionately Enlarged Subarachnoid Space Hydrocephalus (DESH), high convexity/midline tight sulci, and enlarged Sylvian fissures on MRI; **(C)**. Callosal Angle (CA), the angle between the lateral ventricles on coronal MRI images within the anatomical structure of the corpus callosum; **(D)**. z-Evans Index (z-EI), the maximum z-axial length of the frontal horns of the lateral ventricle to the maximum cranial z-axial length at the midline on the coronal plane, precisely located at the anterior commissure, z-EI = A/B. Brain per ventricle ratios (BVRs), BVRs = (B–A)/A; **(E)**. Splenium of the corpus callosum angle (sCCA), the angle between the posterior part of the corpus callosum on axial MRI images; **(F)**. Temporal horns, the width of the temporal horns of the left/right lateral ventricle in the anterior temporal lobe on axial MRI images; **(G)**. Third ventricular width, the maximum width of the third ventricle on axial MRI images. **(H)**. Lateral ventricle posterior angle ratio, the ratio of the maximum width of the posterior horns of the lateral ventricles, and the maximal internal diameter of the skull at the same level employed in axial MRI images.

We conducted a retrospective analysis of clinical data and imaging features in all participants. Preoperative MRI images were evaluated by two seasoned professionals: neuroradiologist Zeng, QZ., and neurosurgeon Gao, W., both of whom were blinded to the clinical characteristics. The final result was the average of their two measurements. If the results differed by more than 5%, the images were reevaluated until consensus was reached. The imaging features include (1) EI, which is the ratio of the maximum width of the frontal horns of the lateral ventricles to the maximal internal diameter of the skull at the same level, as measured in axial MRI images (Figure 2A). (2) DESH, high convexity/midline tight sulci, and enlarged Sylvian fissures on MRI (Figure 2B). (3) CA, the angle between the lateral ventricles on coronal MRI images within the anatomical structure of the corpus callosum (Figure 2C). (4) z-Evans Index (z-EI), the maximum z-axial length of the frontal horns of the lateral ventricle to the maximum cranial z-axial length at the midline on the coronal plane, precisely located at the anterior commissure, z-EI = A/B (Figure 2D). (5) Brain per ventricle ratios (BVRs), The BVRs at the anterior commissure and posterior commissure levels are calculated as the maximum z-axial length of the brain just above the lateral ventricles divided by the maximum length of the lateral ventricle. BVRs = B,A/A (Figure 2D). (6) The splenium of the corpus callosum angle (sCCA), measures the angle between the posterior part of the corpus callosum on axial MRI images (Figure 2E). (7) Temporal horns, measure the width of the temporal horns of the left/right lateral ventricle in the anterior temporal lobe on axial MRI images (Figure 2F). (8) Third ventricular width, measure the maximum width of the third ventricle on axial MRI images (Figure 2G). (9) Lateral ventricle posterior angle ratio, the ratio of the maximum width of the posterior horns of the lateral ventricles, and the maximal internal diameter of the skull at the same level employed in axial MRI images (Figure 2H).

### Statistical analysis

Statistical analysis and visualization were performed using GraphPad Prism software (version 9.0, GraphPad Software, San Diego, CA, United States). In the one-way analysis, the normality of within-group data was initially assessed using the Shapiro-Wilk test, and the homogeneity of variances was evaluated using the F-test. If continuous variables simultaneously met the criteria of normal distribution and homogeneity of variances, a *t*-test was used to analyze intergroup differences. If either the normal distribution or homogeneity of variances criteria were not met, the Mann-Whitney U test was employed. For categorical variables, the chi-square test was used to analyze intergroup differences. In the multivariate analysis, which included the Evans Index, DESH sign and CA as independent variables, a likelihood ratio test with maximum likelihood estimation (forward: LR) was employed. In order to estimate the value of combining radiological features with tap test, DESH or CA were combined with tap test to estimate sensitivity, specificity, and the area under the receiver operating characteristic curve (AUC). Significance levels were denoted as *p < 0.05, **p < 0.01,***p < 0.001 to indicate different levels of statistical significance.

To assess the robustness of the results and evaluate the potential impact of sample size variation across the three centers, a sensitivity analysis was performed. Specifically, we sequentially excluded the data from each center and reanalyzed the main outcomes, including the number of responders and non-responders, tap test results, and key imaging markers (EI, DESH, CA, United States). The consistency of the results across these analyses was used to evaluate the stability of the findings (Supplementary Table 1).

## Results

### Demographic results

In the cohort of 166 enrolled iNPH patients (Table 1), there were 110 males (66.3%) and 56 females (33.7%). The average age of all patients was 71.22 ± 7.08 years. Out of 166 iNPH patients, 96 (57.8%) were responders, and 70 (42.2%) non-responders. In the responder group, the average age was 71.59 ± 6.91 years, with 60 males (62.5%). The mean preoperative lumbar puncture pressure was 129.40 ± 17.79 mmH_2_O, and the average duration of preoperative symptoms was 19.11 ± 7.44 months. A positive tap-test was observed in 61 patients (63.5%). Gait, cognitive, and urinary dysfunction were present in 93 (96.9%), 74 (77.1%), and 76 (79.2%), respectively. In the non-responder group, the average age was 70.71 ± 7.32 years, with 50 males (71.4%). The mean preoperative lumbar puncture pressure was 134.20 ± 14.49 mmH_2_O, and the average duration of preoperative symptoms was 17.69 ± 8.12 months. A positive tap test was observed in 28 patients (40.0%). Gait, cognitive, and urinary dysfunction were present in 67 (95.7%), 55 (78.6%), and 54 (77.1%), respectively. There was a significant statistical difference in the tap-test between the two groups (*p* < 0.01), with a higher positive rate in the tap-test for responders (63.5%) compared to non-responders (40.0%). No statistically significant differences were observed in the comparison of other demographic data between the two groups.

**TABLE 1:** Demographic, clinical feature in idiopathic normal pressure hydrocephalus (iNPH) patients.

	Responders (*n* = 96)	Non-responders (*n* = 70)	*P-*value
Age	71.59 ± 6.91	70.71 ± 7.32	0.43^a^
Sex			0.25^c^
Male	60 (62.5%)	50 (71.4%)	–
Female	36 (37.5%)	20 (28.6%)	–
Preoperative lumbar puncture pressure (mmH_2_O)	129.40 ± 17.79	134.20 ± 14.49	0.06^a^
Disease duration, year	19.11 ± 7.44	17.69 ± 8.12	0.21^b^
Tap test			< 0.01^**^^c^
Positive	61 (63.5%)	28 (40.0%)	–
Negative	35 (36.5%)	42 (60.0%)	–
**Symptoms**			
Motor symptoms	93 (96.9%)	67 (95.7%)	0.70^c^
Cognitive impairment	74 (77.1%)	55 (78.6%)	0.85^c^
Urinary symptoms	76 (79.2%)	54 (77.1%)	0.85^c^
**Comorbidities**			
Cardiovascular diseases	40 (41.7%)	33 (47.1%)	0.53^c^
Endocrine diseases	28 (29.2%)	16 (22.9%)	0.38^c^
Neurological diseases	33 (34.4%)	33 (47.1%)	0.11^c^

^a^*t*-test.

^b^Mann-Whitney U test.

^c^Chi-square test.

^**^p < 0.01.

### The univariate analysis of imaging features

In responders, the preoperative values of magnetic resonance imaging features are as follows (Table 2): EI, mean 0.36 ± 0.04; positive DESH sign, 53 cases (54.6%); CA, mean 71.21 ± 14.62°; z-EI, mean 0.46 ± 0.08; BVRs, mean 0.76 ± 0.20; sCCA, mean 46.25 ± 14.31°; left temporal horn width, mean 8.12 mm ± 2.17 mm; right temporal horn width, mean 7.80 mm ± 1.99 mm; third ventricular width, mean 11.50 mm ± 2.96 mm; and posterior horn ratio of the lateral ventricle, 0.62 ± 0.15. In non-responders, the preoperative values of MRI features are as follows (Table 2): EI, mean 0.35 ± 0.04; positive DESH sign, eight cases (11.6%); CA, mean 76.04 ± 15.06°; z-EI, mean 0.47 ± 0.07; BVRs, mean 0.81 ± 0.18; sCCA, mean 50.24 ± 13.77; left temporal horn width, 7.65 mm ± 2.72 mm; right temporal horn width, mean 7.33 mm ± 2.35 mm; width of the third ventricle, mean 10.94 mm ± 2.81 mm; and posterior horn ratio of the lateral ventricle, 0.60 ± 0.12. The outcomes of VP shunt were more favorable in iNPH patients with a larger EI (*p* = 0.04), positive DESH sign (*p* < 0.001), and a smaller CA (*p* = 0.04).

**TABLE 2:** The univariate analysis of imaging features in 166 idiopathic normal pressure hydrocephalus (iNPH) patients.

	Responders (*n* = 96)	Non-responders (*n* = 70)	*P-*value
EI	0.36 ± 0.04	0.35 ± 0.04	0.04^*^^a^
DESH			0.001^***^^c^
Positive	53	8	–
Negative	43	62	–
CA (°)	71.21 ± 14.62	76.04 ± 15.06	0.04^*^^a^
z-EI	0.46 ± 0.08	0.47 ± 0.07	0.54^a^
BVRs	0.76 ± 0.20	0.81 ± 0.18	0.07^a^
sCCA (°)	46.25 ± 14.31	50.24 ± 13.77	0.05^b^
**Temporal horns (mm)**			
Left	8.12 ± 2.17	7.65 ± 2.72	0.09^b^
Right	7.80 ± 1.99	7.33 ± 2.35	0.07^b^
Third ventricle width (mm)	11.50 ± 2.96	10.94 ± 2.81	0.22^a^
Posterior horn ratio of the lateral ventricle	0.62 ± 0.15	0.60 ± 0.12	0.29^b^

*^a^t*-test.

*^b^*Mann-Whitney U test.

*^c^*Chi-square test. EI, evans index; DESH, disproportionately enlarged subarachnoid space hydrocephalus; CA, callosal angle; z-EI, z-evans index; BVRs, brain-to-ventricle ratios; sCCA, splenium of the corpus callosum angle;

^*^p < 0.05;

^***^p < 0.001.

### The multivariable logistic regression analysis of imaging features

We further included three independent variables, EI, DESH sign and CA into multivariable logistic regression model (Table 3). We found that the DESH sign and CA remained significantly statistically correlated with VP shunt outcome. iNPH patients with preoperative positive DESH sign (OR = 0.09, 95% CI: 0.04–0.22, *p* < 0.001) and a smaller CA (OR = 0.97, 95% CI: 0.95–1.00, *p* = 0.02) had a more favorable shunt response.

**TABLE 3:** The multivariable logistic regression analysis of imaging features in 166 idiopathic normal pressure hydrocephalus (iNPH) patients.

	Wald chi-square value	*P*-value	OR (95% CI)
DESH	28.40	0.001^***^	0.09 (0.04–0.22)
CA	5.45	0.02	0.97 (0.95–1.00)

DESH, disproportionately enlarged subarachnoid space hydrocephalus; CA, callosal angle; OR, odds ratio;

^***^p < 0.001.

### Imaging features combined with tap test for predicting outcome of VP shunt

The results indicate that tap test, DESH sign and CA could predict the outcome of VP shunt in iNPH. To further assess the accuracy of combined model of imaging features with tap test, we calculated sensitivity, specificity, and plotted receiver operating characteristic curves (Table 4).

**TABLE 4:** Sensitivity, specificity, and area under the curve (AUC) of imaging features and combined model in predicting outcome of ventriculoperitoneal (VP) shunt.

	Sensitivity	Specificity	AUC
Tap test	0.64	0.60	0.62
DESH	0.55	0.89	0.72
CA	0.45	0.74	0.60
Tap test + DESH	0.79	0.53	0.66
Tap test + CA	0.85	0.51	0.68
Tap test + DESH + CA	0.77	0.67	0.72

AUC, area under the curve; DESH, disproportionately enlarged subarachnoid space hydrocephalus; CA, callosal angle.

Firstly, we calculated the optimal cut-off of CA for predicting shunt outcomes in iNPH using Youden index, which was found to be 68.7°. When using the individual metrics of tap test, DESH, and CA to predict the outcome of VP shunt, the sensitivity were 0.64, 0.55, and 0.45, respectively; the specificity were 0.60, 0.89, and 0.74, respectively; and the AUC were 0.62, 0.72, and 0.60, respectively. These results indicate that imaging features with higher specificity could improve the screening of iNPH patients who do not respond to VP shunt. However, this increase in specificity is accompanied by a decrease in sensitivity, implying that fewer responders could be identified.

We combined DESH and CA with the tap test based on the following criteria: if the tap test result was negative but DESH was positive, the combined tap test + DESH indicator was also considered positive, which is consistent with the criteria for diagnosing probable iNPH in the Japanese guidelines (Nakajima et al., 2021). The same criteria were applied to the combination of the tap test and CA. By using tap test combined with DESH or CA, the sensitivity was 0.79 and 0.85, respectively; the specificity was 0.53 and 0.51, respectively, and the AUC was 0.66 and 0.68, respectively. Although sensitivity increased significantly, specificity had a remarkable decrease, which implied that while more responders (10 in tap test + DESH and 16 in tap test + CA) are identified, whereas there is a significant increase in the misdiagnosis of patients as positive (5 in tap test + DESH and 6 in tap test + CA). This indicates that merely using a single imaging indicator to increase the sensitivity of the tap test might be too permissive.

**FIGURE 3:**
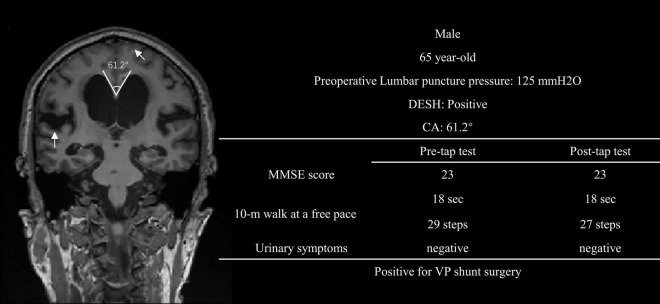
A representative case of negative tap test with positive DESH and smaller CA, who benefited from VP shunt surgery. DESH, subarachnoid Space Hydrocephalus; CA, callosal Angle; MMSE, mini-mental state examination; VP shunt surgery, ventriculoperitoneal shunting surgery.

In the clinical practice, we observed that some patients with negative tap test results but positive DESH and a smaller CA could benefit from shunt surgery (Figure 3 showcases a representative case). Informed by these clinical insights, we introduced a novel predictive model: Based on the tap test results, if the tap is positive but both DESH and CA are negative, the model is classified as negative. Conversely, if the tap is negative but both DESH and CA are positive, the model is considered as positive. The sensitivity, specificity, and AUC of combined model were 0.77, 0.67 and 0.72, respectively. The imaging features combined with tap test predictive model significantly improved the sensitivity, specificity, and AUC compared to tap test.

## Discussion

This study primarily investigates the imaging features combined by tap test to identify iNPH patients who respond to VP shunt surgery. Given the poor understanding of the pathological mechanisms underlying iNPH, the imaging features utilized to predict shunt outcomes in iNPH predominantly focus on alterations in brain structural features. Previous studies have documented that brain structural imaging features can effectively distinguish iNPH from other conditions leading to secondary ventricular enlargement (Vlasák et al., 2022; Luca et al., 2023). There are few imaging features to be reliable candidates as a predictor for VP shunt surgery (Kojoukhova et al., 2015). The DESH sign currently stands out as the most predominantly utilized imaging feature as recommended by the Japanese guidelines (Mori et al., 2012) and the European guidelines (Relkin et al., 2005), which is consistent with our results. However, the prevalence of the DESH sign only showed approximately in 36%–44% iNPH patients according to Agerskov et al. (2019) and Park et al. (2021), and 36.7% in our dataset. Additionally, a significant challenge in the clinical application of the DESH sign is the absence of a standardized quantitative assessment method, often leading to subjective evaluations by clinicians. Recently, Shinoda et al. (2017) developed a clinical imaging scales that facilitates the quantification of the DESH index, showing a highly significant statistical correlation with responders.

The results reveal a significant statistical difference in CA between responders and non-responders. The CA has been previously reported as a potential imaging feature for predicting VP shunt outcomes in iNPH (Pyrgelis et al., 2022; Thavarajasingam et al., 2022). A retrospective analysis of 109 iNPH patients who underwent shunt surgery suggested that CA could effectively distinguish shunt responders from non-responders (Virhammar et al., 2014b). Shunt responders exhibited a significantly smaller preoperative CA (59° versus 68°). The CA of 63° was determined as the cut-off value with the highest accuracy in prediction, achieving a sensitivity of 0.67 and specificity of 0.65. In our study, the optimal cut-off value for CA was similar at 68.7°, yielding a sensitivity of 0.45 and specificity of 0.74. While this represents an improvement in specificity compared to previous studies, there is a decline in sensitivity. Future research with larger sample sizes will be necessary to establish the optimal threshold of CA for distinguishing shunt responders from non-responders.

The clinical guideline included tap test and DESH sign as a criterion to make the diagnosis of iNPH. If a patient meets the criteria for suspected iNPH and presents a positive DESH sign or tap test, the diagnosis could be further classified as probable iNPH (Mori et al., 2012). Nevertheless, the positive rate of the tap test and DESH sign are relatively low, and patients lacking the positive tap test result or DESH sign may still respond to shunt treatment. Identifying individuals with potential shunt improvement among those patients is crucial for enhancing the accuracy of clinical practice related to shunt outcomes. In our study, the incorporation of both DESH sign and CA into an imaging combined model demonstrated an enhanced ability to predict shunt treatment outcomes in iNPH.

When DESH or CA was separately incorporated into tap test, the detection rate of positive patients (sensitivity) was remarkably enhanced. However, this enhancement comes at the cost of significantly reduced specificity, resulting in an increased number of false positives. To address this limitation, we developed a novel model integrating the tap test with both DESH and CA. This comprehensive approach not only enhances sensitivity (from 0.64 to 0.77) but also improves specificity (from 0.60 to 0.67), offering a more robust diagnostic framework.

Previous studies also suggested other imaging features for predicting the efficacy of shunt treatment in iNPH. The EI, with a cut-off of > 0.3, is frequently employed to evaluate the extent of ventricular enlargement in patients. Nevertheless, the correlation between the degree of ventricular enlargement and the shunt prognosis in iNPH is not thoroughly understood. Subramanian et al. (2021), discovered that the EI could predict cognitive improvement in iNPH patients after shunt treatment (a higher Evans Index associated with better cognitive improvement, *p* < 0.01), but it could not predict overall symptom improvement 1 year after shunting. Chen et al. (2022), suggest that the EI is statistically correlated with the severity of clinical symptoms in iNPH patients but is unable to predict the efficacy of shunt treatment 1 year post-surgery. Moreover, several studies have indicated that the EI does not exhibit a significant correlation with the shunt prognosis in iNPH (Virhammar et al., 2014a; Kojoukhova et al., 2015; Agerskov et al., 2019), which aligns with the findings of our study. Earlier research has identified a notable correlation between the enlargement of the third and fourth ventricles and the severity of gait disturbances in iNPH patients. This implies that structures surrounding the ventricles may play a role in the progression of clinical symptoms in iNPH (Virhammar et al., 2014a; Lotan et al., 2022). Nonetheless, there is limited research and evidence concerning the correlation between the third ventricle and the shunt outcomes in iNPH. Saito et al. (2020), discovered that a decrease in the volume of the third ventricle was associated with cognitive improvement after shunting, indicating that the width of the third ventricle could potentially serve as a marker for shunt prognosis. A study by Soon et al. (2021), suggested that the width of the third ventricle not only efficiently differentiated between patients with and without hydrocephalus but also predicted the efficacy of shunt treatment. Nevertheless, the majority of studies contend that the width of the third ventricle does not exhibit a significant statistical correlation with the shunt prognosis in iNPH (Virhammar et al., 2014a; Agerskov et al., 2019), with is consistent with our results. While Laticevschi et al. (2021) proposed that patients with smaller temporal horns had a higher positive rate in tap-test, our study results reveal no significant statistical correlation between the temporal horns and shunt prognosis in iNPH. This is in line with the findings of Kojoukhova et al. (2015), The sCCA, introduced by Chan et al. (2021), is another imaging feature used to assess the degree of lateral ventricular expansion. It was initially demonstrated to effectively differentiate between iNPH patients and non-iNPH patients, including normal controls, Alzheimer’s disease, and Parkinson’s disease. The diagnostic accuracy of the sCCA alone was found to be higher than that of EI and CA (Chan et al., 2021). However, there is currently no other research confirming that the sCCA can predict the efficacy of shunt treatment in iNPH.

## Limitation

This study has some limitations. First, the MMSE has limited sensitivity to detect subtle cognitive changes, which may affect the evaluation of outcomes. Second, gait was re-evaluated 24–72 h after lumbar puncture, which could have delayed the detection of earlier improvements and reduced the sensitivity of tap test. Finally, as a multicenter retrospective study, the contribution of sample sizes from different centers may be uneven, potentially resulting in certain centers disproportionately influencing the research outcomes. Variations in MRI acquisition parameters across centers could introduce inconsistencies, potentially affecting the measurement consistency of imaging biomarkers.

## Conclusion

Disproportionately Enlarged Subarachnoid Space Hydrocephalus sign and CA could serve as potential imaging features for predicting the outcomes of VP shunt surgery, as evidenced by our multicenter retrospective study on iNPH cases. In comparison to the tap test, the combination of DESH and CA with tap test significantly improves both sensitivity and specificity in screening VP shunt responders.

## Data Availability

The raw data supporting the conclusions of this article will be made available by the authors, without undue reservation.

## References

[B1] Agerskov S. Wallin M. Hellström P. Ziegelitz D. Wikkelsö C. Tullberg M. (2019). Absence of disproportionately enlarged subarachnoid space hydrocephalus, a sharp callosal angle, or other morphologic mri markers should not be used to exclude patients with idiopathic normal pressure hydrocephalus from shunt surgery. *AJNR Am. J. Neuroradiol.* 40 74–79. 10.3174/ajnr.A5910 30523139 PMC7048608

[B2] Andrén K. Wikkelsø C. Tisell M. Hellström P. (2014). Natural course of idiopathic normal pressure hydrocephalus. *J. Neurol. Neurosurg. Psychiatry* 85 806–810. 10.1136/jnnp-2013-306117 24292998

[B3] Carlsen J. F. Munch T. N. Hansen A. E. Hasselbalch S. G. Rykkje A. M. (2022). Can preoperative brain imaging features predict shunt response in idiopathic normal pressure hydrocephalus? A prisma review. *Neuroradiology* 64 2119–2133. 10.1007/s00234-022-03021-9 35871239

[B4] Chan L. L. Chen R. Li H. Lee A. J. Y. Go W. Y. Lee W. (2021). The splenial angle: A novel radiological index for idiopathic normal pressure hydrocephalus. *Eur. Radiol.* 31 9086–9097. 10.1007/s00330-021-07871-4 33991224 PMC8589785

[B5] Chen J. He W. Zhang X. Lv M. Zhou X. Yang X. (2022). Value of MRI-based semi-quantitative structural neuroimaging in predicting the prognosis of patients with idiopathic normal pressure hydrocephalus after shunt surgery. *Eur. Radiol.* 32 7800–7810. 10.1007/s00330-022-08733-3 35501572 PMC9668801

[B6] Craven C. L. Toma A. K. Mostafa T. Patel N. Watkins L. D. (2016). The predictive value of DESH for shunt responsiveness in idiopathic normal pressure hydrocephalus. *J. Clin. Neurosci.* 34 294–298. 10.1016/j.jocn.2016.09.004 27692614

[B7] Junkkari A. Sintonen H. Danner N. Jyrkkänen H. K. Rauramaa T. Luikku A. J. (2021). 5-Year health-related quality of life outcome in patients with idiopathic normal pressure hydrocephalus. *J. Neurol.* 268 3283–3293. 10.1007/s00415-021-10477-x 33651154 PMC8357651

[B8] Kojoukhova M. Koivisto A. M. Korhonen R. Remes A. M. Vanninen R. Soininen H. (2015). Feasibility of radiological markers in idiopathic normal pressure hydrocephalus. *Acta Neurochir.* 157 1709–1719. 10.1007/s00701-015-2503-8 26190755

[B9] Laticevschi T. Lingenberg A. Armand S. Griffa A. Assal F. Allali G. (2021). Can the radiological scale “iNPH Radscale” predict tap test response in idiopathic normal pressure hydrocephalus? *J. Neurol. Sci.* 420:117239. 10.1016/j.jns.2020.117239 33278661

[B10] Lotan E. Damadian B. E. Rusinek H. Griffin M. Ades-Aron B. Lu N. (2022). Quantitative imaging features predict spinal tap response in normal pressure hydrocephalus. *Neuroradiology* 64 473–481. 10.1007/s00234-021-02782-z 34417636

[B11] Luca A. Donzuso G. Mostile G. Terranova R. Cicero C. E. Nicoletti A. (2023). Brain linear measurements for differentiating normal pressure hydrocephalus from Alzheimer’s disease: An exploratory study. *Eur. J. Neurol.* 30 2849–2853. 10.1111/ene.15904 37265410

[B12] Martín-Láez R. Caballero-Arzapalo H. López-Menéndez L. Á Arango-Lasprilla J. C. Vázquez-Barquero A. (2015). Epidemiology of idiopathic normal pressure hydrocephalus: A systematic review of the literature. *World Neurosurg.* 84 2002–2009. 10.1016/j.wneu.2015.07.005 26183137

[B13] Mihalj M. Dolić K. Kolić K. Ledenko V. (2016). CSF tap test — Obsolete or appropriate test for predicting shunt responsiveness? A systemic review. *J. Neurol. Sci.* 362 78–84. 10.1016/j.jns.2016.01.028 26944123

[B14] Miyajima M. Kazui H. Mori E. Ishikawa M. (2016). One-year outcome in patients with idiopathic normal-pressure hydrocephalus: Comparison of lumboperitoneal shunt to ventriculoperitoneal shunt. *JNS* 125 1483–1492. 10.3171/2015.10.JNS151894 26871203

[B15] Mori E. Ishikawa M. Kato T. Kazui H. Miyake H. Miyajima M. (2012). Guidelines for management of idiopathic normal pressure hydrocephalus: Second edition. *Neurol. Med. Chir.* 52 775–809. 10.2176/nmc.52.775 23183074

[B16] Nakajima M. Yamada S. Miyajima M. Ishii K. Kuriyama N. Kazui H. (2021). Guidelines for management of idiopathic normal pressure hydrocephalus (Third Edition): Endorsed by the Japanese society of normal pressure hydrocephalus. *Neurol. Med. Chir.* 61 63–97. 10.2176/nmc.st.2020-0292 33455998 PMC7905302

[B17] Park H. Y. Park C. R. Suh C. H. Kim M. J. Shim W. H. Kim S. J. (2021). Prognostic utility of disproportionately enlarged subarachnoid space hydrocephalus in idiopathic normal pressure hydrocephalus treated with ventriculoperitoneal shunt surgery: A systematic review and meta-analysis. *AJNR Am. J. Neuroradiol.* 42 1429–1436. 10.3174/ajnr.A7168 34045302 PMC8367621

[B18] Pyrgelis E.-S. Paraskevas G. P. Constantinides V. C. Boufidou F. Velonakis G. Stefanis L. (2022). Callosal angle sub-score of the radscale in patients with idiopathic normal pressure hydrocephalus is associated with positive tap test response. *JCM* 11:2898. 10.3390/jcm11102898 35629023 PMC9143138

[B19] Relkin N. Marmarou A. Klinge P. Bergsneider M. Black P. (2005). Diagnosing idiopathic normal-pressure hydrocephalus. *Neurosurgery* 57 S2-4–S2-16. 10.1227/01.NEU.0000168185.29659.C5. 16160425

[B20] Rydja J. Eleftheriou A. Lundin F. (2021). Evaluating the cerebrospinal fluid tap test with the Hellström iNPH scale for patients with idiopathic normal pressure hydrocephalus. *Fluids Barriers CNS* 18:18. 10.1186/s12987-021-00252-5 33827613 PMC8025497

[B21] Saito A. Kamagata K. Ueda R. Nakazawa M. Andica C. Irie R. (2020). Ventricular volumetry and free-water corrected diffusion tensor imaging of the anterior thalamic radiation in idiopathic normal pressure hydrocephalus. *J. Neuroradiol.* 47 312–317. 10.1016/j.neurad.2019.04.003 31034894

[B22] Shinoda N. Hirai O. Hori S. Mikami K. Bando T. Shimo D. (2017). Utility of MRI-based disproportionately enlarged subarachnoid space hydrocephalus scoring for predicting prognosis after surgery for idiopathic normal pressure hydrocephalus: Clinical research. *J. Neurosurg.* 127 1436–1442. 10.3171/2016.9.JNS161080 28156249

[B23] Soon S. X. Y. Kumar A. A. Tan A. J. L. Lo Y. T. Lock C. Kumar S. (2021). The impact of multimorbidity burden, frailty risk scoring, and 3-directional morphological indices vs. testing for CSF responsiveness in normal pressure hydrocephalus. *Front. Neurosci.* 15:751145. 10.3389/fnins.2021.751145 34867163 PMC8636813

[B24] Subramanian H. E. Fadel S. A. Matouk C. C. Zohrabian V. M. Mahajan A. (2021). The utility of imaging parameters in predicting long-term clinical improvement after shunt surgery in patients with idiopathic normal pressure hydrocephalus. *World Neurosurg.* 149 e1–e10. 10.1016/j.wneu.2021.02.108 33662608

[B25] Thavarajasingam S. G. El-Khatib M. Vemulapalli K. Iradukunda H. A. S. Vishnu K. Borchert R. (2022). Radiological predictors of shunt response in the diagnosis and treatment of idiopathic normal pressure hydrocephalus: A systematic review and meta-analysis. *Acta Neurochir.* 165 369–419. 10.1007/s00701-022-05402-8 36435931 PMC9922237

[B26] Virhammar J. Laurell K. Cesarini K. G. Larsson E.-M. (2014a). Preoperative prognostic value of MRI findings in 108 patients with idiopathic normal pressure hydrocephalus. *AJNR Am. J. Neuroradiol.* 35 2311–2318. 10.3174/ajnr.A4046 25012669 PMC7965311

[B27] Virhammar J. Laurell K. Cesarini K. G. Larsson E.-M. (2014b). The callosal angle measured on MRI as a predictor of outcome in idiopathic normal-pressure hydrocephalus: Clinical article. *JNS* 120 178–184. 10.3171/2013.8.JNS13575 24074491

[B28] Vlasák A. Gerla V. Skalický P. Mládek A. Sedlák V. Vrána J. (2022). Boosting phase-contrast MRI performance in idiopathic normal pressure hydrocephalus diagnostics by means of machine learning approach. *Neurosurg. Focus* 52:E6. 10.3171/2022.1.FOCUS21733 35364583

[B29] Walchenbach R., Geiger E. *The Value of Temporary External Lumbar CSF Drainage in Predicting the Outcome of Shunting on Normal Pressure Hydrocephalus.*.

[B30] Wikkelsø C. Hellström P. Klinge P. M. Tans J. T. J. (2013). The European iNPH multicentre study on the predictive values of resistance to CSF outflow and the CSF tap test in patients with idiopathic normal pressure hydrocephalus. *J. Neurol. Neurosurg. Psychiatry* 84 562–568. 10.1136/jnnp-2012-303314 23250963

[B31] Wu E. M. El Ahmadieh T. Y. Kafka B. Caruso J. P. Neeley O. J. Plitt A. R. (2020). Clinical outcomes of normal pressure hydrocephalus in 116 patients: Objective versus subjective assessment. *J. Neurosurg.* 132 1757–1763. 10.3171/2019.1.JNS181598 30978684

