# Supplementary material for: Preoperative imaging biomarkers combined with tap test for predicting shunt surgery outcome in idiopathic normal pressure hydrocephalus: a multicenter retrospective study
Source: Front Aging Neurosci. 2025 Feb 27;17:1509493. doi: 10.3389/fnagi.2025.1509493 (PMC11903477; doi:10.3389/fnagi.2025.1509493)
Supplement: Supplementary file 1 [file Table_1.docx]

**Supplementary Table 1.** Sensitivity analysis of the data.

|  | Group A vs B  (*P* value) | Group A vs C  (*P* value) | Group A vs D  (*P* value) |
| --- | --- | --- | --- |
| **Tap test** | 0.87 | 0.28 | 0.09 |
| **Evans index** | 0.68 | 0.69 | 0.93 |
| **DESH** | 0.99 | 0.84 | 0.68 |
| **CA** | 0.96 | 0.13 | 0.10 |
| **Outcome** | 0.98 | 0.63 | 0.33 |

DESH, Disproportionately enlarged subarachnoid space hydrocephalus; CA, Callosal angle;

Group A contains data for centers 1,2, and 3;

Group B contains data for centers 1 and 2;

Group C contains data for centers 1 and 3;

Group D contains data for centers 2 and 3;
